# Draft genome sequence and characterization of commensal *Escherichia coli* strain BG1 isolated from bovine gastro-intestinal tract

**DOI:** 10.1186/s40793-017-0272-0

**Published:** 2017-10-10

**Authors:** Audrey Segura, Pauline Auffret, Christophe Klopp, Yolande Bertin, Evelyne Forano

**Affiliations:** 1 Université Clermont Auvergne, INRA, MEDIS, F-63000 Clermont-Ferrand, France; 2 Plateforme Bioinformatique Toulouse, Midi-Pyrénées UBIA, INRA, Auzeville Castanet-Tolosan, France

**Keywords:** *Escherichia coli*, Commensal, Bovine, Gastro-intestinal tract, Whole genome sequencing, Virulence factors, Ethanolamine

## Abstract

*Escherichia coli* is the most abundant facultative anaerobic bacteria in the gastro-intestinal tract of mammals but can be responsible for intestinal infection due to acquisition of virulence factors. Genomes of pathogenic *E. coli* strains are widely described whereas those of bovine commensal *E. coli* strains are very scarce. Here, we report the genome sequence, annotation, and features of the commensal *E. coli* BG1 isolated from the gastro-intestinal tract of cattle. Whole genome sequencing analysis showed that BG1 has a chromosome of 4,782,107 bp coding for 4465 proteins and 97 RNAs. *E. coli* BG1 belonged to the serotype O159:H21, was classified in the phylogroup B1 and possessed the genetic information encoding “virulence factors” such as adherence systems, iron acquisition and flagella synthesis. A total of 12 adherence systems were detected reflecting the potential ability of BG1 to colonize different segments of the bovine gastro-intestinal tract. *E. coli* BG1 is unable to assimilate ethanolamine that confers a nutritional advantage to some pathogenic *E. coli* in the bovine gastro-intestinal tract. Genome analysis revealed the presence of i) 34 amino acids change due to non-synonymous SNPs among the genes encoding ethanolamine transport and assimilation, and ii) an additional predicted alpha helix inserted in cobalamin adenosyltransferase, a key enzyme required for ethanolamine assimilation. These modifications could explain the incapacity of BG1 to use ethanolamine. The BG1 genome can now be used as a reference (control strain) for subsequent evolution and comparative studies.

**Electronic supplementary material:**

The online version of this article (10.1186/s40793-017-0272-0) contains supplementary material, which is available to authorized users.

## Introduction

10.1601/nm.3093 is a common inhabitant of the gastro-intestinal tract of humans and animals [1]. In particular, 10.1601/nm.3093 is typically the most common facultative anaerobe in the lower intestine of mammals and its presence in the environment is usually considered to reflect fecal contamination [1, 2]. The 10.1601/nm.3093 population is multiclonal and fluctuates in its predominance depending on diet, exposure to antibiotics or interactions with the host endogenous microbiota [1].

The intestinal microbiota predominantly comprises strict anaerobic bacteria, especially in the colon. 10.1601/nm.3093 exists in a symbiotic relationship with strict anaerobes: 10.1601/nm.3093 ferments monosaccharides generated by the degradation of polysaccharides by anaerobes (10.1601/nm.3093 being unable to synthesize the necessary hydrolase enzymes) and in turn, 10.1601/nm.3093 is able to consume oxygen and therefore to favor the strict anaerobe multiplication by creating a more anaerobic environment [2, 3]. Similarly, the host-10.1601/nm.3093 relationship is mutualistic: the intestinal environment promotes efficient 10.1601/nm.3093 survival and multiplication and in turn, the 10.1601/nm.3093 population produces vitamins K and B12, which are required by mammalian hosts, and competitively excludes pathogens from the host intestinal tract [2]. 10.1601/nm.3093 strains are able to colonize various locations in the mammalian gastro-intestinal tract, but they are mainly found on the mucus layer used by 10.1601/nm.3093 as an essential nutritional source [4]. Successful colonization of the gastro-intestinal tract by 10.1601/nm.3093 depends upon several factors: competition for nutrients with the autochthonous microbiota, production of adhesins to bring the bacteria closer to the epithelia, penetration of the mucus layer, rapid growth and biofilm formation ability [1, 2, 4]. If 10.1601/nm.3093 growth does not exceed the turnover rate of the mucus layer, the bacterial cells are sloughed off into the intestine lumen and then eliminated in the feces [4]. Therefore, 10.1601/nm.3093 must display metabolic flexibility and grow in biofilm in order to succeed in this very competitive biotope [4].

Although considered as commensal in the mammalian gut, 10.1601/nm.3093 also causes a broad range of intestinal or extra-intestinal diseases due to the acquisition of mobile genetic elements encoding virulence factors. Among pathogenic 10.1601/nm.3093, STEC is the major food-borne pathogen responsible for hemorrhagic colitis and hemolytic uremic syndrome [5]. In particular, a STEC strain subgroup EHEC belonging mostly to the serotype O157:H7 is responsible for serious public health concern and financial burden [5]. STEC strains are mainly transmitted to humans through contaminated meat or unpasteurized milk consumption [6]. It is of interest to note that healthy ruminants, mainly cattle, are the principal reservoir for 10.1601/nm.3093 O157:H7 strains, but cattle lack the Shiga-toxin vascular receptor, which explains why they are Shiga-toxin tolerant [6].

The cost of whole genome sequencing has decreased drastically and it is now possible to sequence a large number of isolates and use bioinformatic approaches to extract strain relatedness and gene carriage data. 10.1601/nm.3093 strains involved in human infections have been extensively studied and many whole genome sequences of 10.1601/nm.3093 associated with human illness are now available, allowing exploration of pathogenicity processes and identification of virulence factors. Due to cattle STEC dissemination, a significant number of whole genomes of 10.1601/nm.3093 O157:H7 strains isolated from bovine have also been sequenced. While previous genome sequencing efforts with commensal intestinal 10.1601/nm.3093 have focused on human strains [7–9], such data are scarce concerning commensal 10.1601/nm.3093 strains isolated from the bovine gastro-intestinal tract. It would be valuable to have recent and reliable genomic data on bovine commensal strains to be used as reference genomes.

In this study, we report the draft genome sequence and preliminary functional annotation of the commensal 10.1601/nm.3093 strain BG1 isolated from the digestive tract of a cow. The strain BG1 has been previously included in studies concerning the adaptation of pathogenic and commensal 10.1601/nm.3093 strains in the bovine gastro-intestinal tract [10, 11]. This study aimed to characterize the genomic features of the BG1 strain in order to provide information for future genomic scale (whole genome) comparative analyses. The organism is not part of a larger genomic survey project.

## Organism information

### Classification and features

**Fig. 1:**
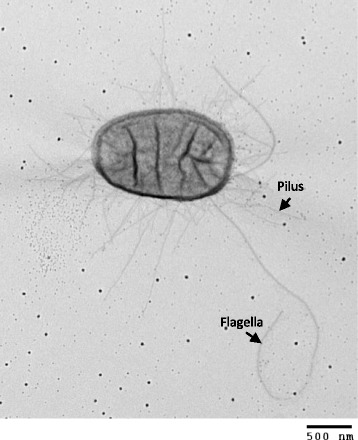
Transmission electron micrograph of *E. coli* BG1. The strain BG1 is a rod-shaped bacteria with a length of 1.5–2 μm and a diameter of 1 μm. It moves via peritrichous flagella. The magnification rate is 20,000×. The scale bar indicates 500 nm.

As described for the genus 10.1601/nm.3092, 10.1601/nm.3093 BG1 is a Gram-negative, rod-shaped bacterium belonging to the 10.1601/nm.3091 family (Table 1). 10.1601/nm.3093 is a facultative anaerobe that is motile by means of flagella (Fig. 1). 10.1601/nm.3093 strains are typically able to grow over a wide temperature range (15–48 °C) with optimum growth from 37 to 42 °C and within a pH range of 5.5–8.0 (the best growth occurs at pH 7) [1] (Table 1). Like typical members of the 10.1601/nm.3093 species, the commensal strain BG1 utilizes D-glucose, D-mannitol, L-rhamnose, D-saccharose, D-melibiose and L-arabinose. Unlike most pathogenic O157:H7 EHEC strains, the strain BG1 is able to use sorbitol as a carbon source. In addition, 10.1601/nm.3093 BG1 is positive for arginine dihydrolase, ornithine decarboxylase, β-galactosidase and indole production.

**Table 1:** Classification and general features of *E. coli* BG1 [58]

MIGS ID	Property	Term	Evidence code^a^
	Classification	Domain *Bacteria*	TAS [59]
		Phylum *Proteobacteria*	TAS [60]
		Class *Gammaproteobacteria*	TAS [61, 62]
		Order “*Enterobacteriales”*	TAS [63]
		Family *Enterobacteriaceae*	TAS [64, 65]
		Genus *Escherichia*	TAS [66, 67]
		Species *Escherichia coli*	TAS [66, 67]
	Gram stain	Negative	IDA, TAS [1]
	Cell shape	Rod	IDA, TAS [1]
	Motility	Motile	TAS [1]
	Sporulation	None	TAS [1]
	Temperature range	*≈ 15–48 °C*	TAS [1]
	Optimum temperature	*37–42 °C*	TAS [1]
	pH range; Optimum	*5.5–8.0; 7*	TAS [1]
	Carbon source	Carbohydrates, amino acids	IDA, TAS [1]
MIGS-6	Habitat	Bovine digestive tract	IDA
MIGS-6.3	Salinity	Not reported	
MIGS-22	Oxygen requirement	Facultative anaerobe	TAS [1]
MIGS-15	Biotic relationship	Commensalism	IDA
MIGS-14	Pathogenicity	Non-pathogenic	
MIGS-4	Geographic location	France	
MIGS-5	Sample collection	January 14, 2009	
MIGS-4.1	Latitude	Not reported	
MIGS-4.2	Longitude	Not reported	
MIGS-4.4	Altitude	Not reported	

^a^Evidence codes – *IDA* Inferred from Direct Assay; *TAS* Traceable Author Statement (i.e., a direct report exists in the literature); *NAS* Non-traceable Author Statement (i.e., not directly observed for the living, isolated sample, but based on a generally accepted property for the species, or anecdotal evidence). These evidence codes are from the Gene Ontology project [68]

In silico serotyping using SerotypeFinder (version 1.1) [12] revealed that 10.1601/nm.3093 BG1 belongs to the serotype O159:H21. The whole genome of 10.1601/nm.3093 BG1 lacked all the genes encoding antimicrobial resistance screened using ResFinder (version 2.1) [13]. 10.1601/nm.3093 strains can be divided into different phylogroups (A, B1, B2, D and E) commonly used to investigate the evolution and diversity of 10.1601/nm.3093 strains [14]. Phylogrouping was performed in silico using the quadruplex method described by Clermont et al. [14] and the primersearch program from the EMBOSS open software suite [15]. 10.1601/nm.3093 BG1 belongs to the phylogroup B1, which is commonly distributed among both bovine commensal and human pathogenic 10.1601/nm.3093 strains [16, 17].

**Fig. 2:**
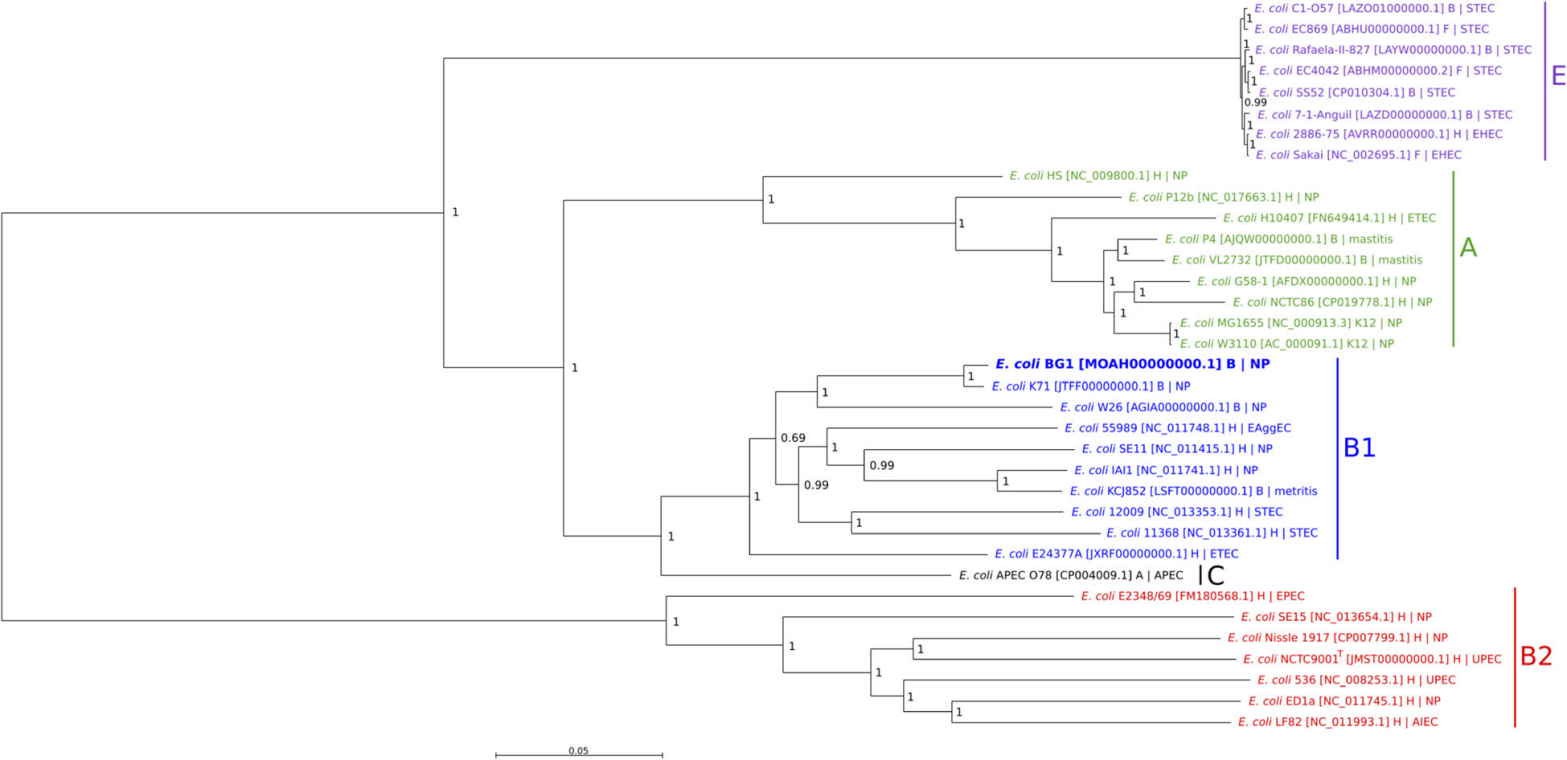
Phylogenetic tree highlighting the position of *E. coli* BG1 relative to other *E. coli* strains. The whole genome SNP based phylogeny was established with CSI phylogeny 1.4 [28] using the genome of K71 as a reference and standard input parameters. The tree was midpoint rooted and plotted using Seaview (version 4.6.1) [56]. Each strain is identified as H (Human), B (Bovine), A (Avian), F (Food) or K12 (Laboratory strain), and its clinical or non-pathogenic (NP) characteristic is specified.

A whole genome phylogenetic analysis based on single nucleotide polymorphism (SNP) differences in 10.1601/nm.3093 BG1, bovine and human commensal 10.1601/nm.3093 strains, bovine pathogenic 10.1601/nm.3093 strains and bovine O157:H7 STEC strains (Additional file 1: Table S1) was conducted using CSI Phylogeny (version 1.4) [18]. Published 10.1601/nm.3093 genomes representing different 10.1601/nm.3093 pathotypes were selected for genomic comparison (Additional file 1: Table S1). In addition, two reference 10.1601/nm.3093 strains, one of which is the 10.1601/nm.3093 type strain (10.1601/strainfinder?urlappend=%3Fid%3DNCTC+9001 ^T^), were also included in this study. As shown in Fig. 2, the bacterial strains were clustered according to the phylogroup classification: BG1 was clustered with commensal and pathogenic 10.1601/nm.3093 strains belonging to phylogroup B1 (EHEC, STEC, ETEC, EAEC, APEC and 10.1601/nm.3093 responsible for postpartum metritis in dairy cows). The closest relative strains to BG1 were 10.1601/nm.3093 K71 isolated from the environment of a cow shed and 10.1601/nm.3093 W26 isolated from bovine feces, both of which belong to the phylogroup B1 (Fig. 2). In contrast, BG1 was more distantly clustered to pathogenic bovine and human 10.1601/nm.3093 strains (Fig. 2). However, 10.1601/nm.3093 KCJ852 (phylogroup B1), which is responsible for metritis, was more closely clustered to BG1 than the P4 and VL2732 strains associated with bovine mastitis (phylogroup A) (Fig. 2). It is of interest to note that i) the bovine 10.1601/nm.3093 strains of commensal origin (BG1, K71 and W26) were distantly related to bovine STEC O157:H7 strains (phylogroup E) and ii) the SNP-based phylogeny analysis failed to cluster the commensal 10.1601/nm.3093 strains according to their human or animal origin.

## Genome sequencing information

### Genome project history

Bovine commensal 10.1601/nm.3093 strains are poorly documented. Therefore, the 10.1601/nm.3093 BG1 strain was selected for genome sequencing to provide valuable genetic information for future genomic scale (whole genome) comparative analysis. 10.1601/nm.3093 BG1 has been used as a reference strain in studies related to carbon and nitrogen nutrition of 10.1601/nm.3093 strains in the bovine gastro-intestinal tract [10, 11]. The strain BG1 was isolated from the small intestine content of a cow at the slaughterhouse in January 2009. The animal was raised and slaughtered in accordance with the guidelines of the local ethics committee and current INRA (National Institute for Agricultural Research) ethical guidelines for animal welfare (Slaughterhouse Permit number: 63,345,001). The bovine intestinal samples were collected after the slaughter of animals required for experiments specifically approved by the “Comité d’éthique en matière d’expérimentation animale en Auvergne” (Permit number: CE22-08) in the experimental slaughterhouse of the “Herbipole”, INRA Saint-Genès-Champanelle, France. The Whole Genome Shotgun project was deposited at DDBJ/ENA/GenBank under the accession MOAH00000000 (Oct 31, 2016). A summary of the sequencing project information is provided in Table 2.

**Table 2:** Genome sequencing project information for *E. coli* BG1

MIGS ID	Property	Term
MIGS 31	Finishing quality	High quality draft
MIGS-28	Libraries used	Paired ends library
MIGS 29	Sequencing platforms	Illumina MiSeq
MIGS 31.2	Fold coverage	127×
MIGS 30	Assemblers	SPAdes version 3.1.1
MIGS 32	Gene calling method	PROKKA version 1.10
	Locus Tag	BLX34
	Genbank ID	MOAH00000000
	GenBank Date of Release	2017–02-24
	GOLD ID	
	BIOPROJECT	PRJNA351833
MIGS 13	Source Material Identifier	BG1
	Project relevance	Commensal *E. coli* (control strain)

### Growth conditions and genomic DNA preparation

10.1601/nm.3093 BG1 was inoculated in Luria-Bertani broth from a single colony and incubated at 37 °C with shaking (200 rpm) to early stationary phase. The bacterial suspension was then centrifuged (10,000 *g* for 15 min) and the total DNA was extracted from the bacterial pellet using the DNeasy Blood and Tissue Kit following the manufacturer’s recommendations (Qiagen). DNA was quantified using a Nanodrop spectrophotometer and DNA integrity was electrophoretically verified by ethidium bromide staining.

### Genome sequencing and assembly

Whole genome sequencing was performed at the GeT-PlaGe core facility (INRA Toulouse, France). DNA-seq libraries were prepared according to Illumina’s protocols using the Illumina TruSeq Nano DNA LT Library Prep Kit. Briefly, DNA was fragmented by sonication using a Covaris M220 and adapters were ligated to be sequenced. Eight cycles of PCR were applied to amplify libraries. Library quality was assessed using the Agilent Bioanalyzer and libraries were quantified by qPCR using the Kapa Library Quantification Kit. DNA-seq experiments were performed on an Illumina MiSeq using a paired-end read length of 2 × 250 bp with the Illumina MiSeq Reagent Kits v2. The raw reads were stored in ng6 [19] and quality was checked using fastqc [20]. They were assembled with SPAdes (version 3.1.1) [21] using standard parameters.

### Genome annotation

The assembled contigs were annotated with Prokka (version 1.10) [22] using standard parameters. Predicted genes were also assigned to functional categories of Clusters of Orthologous Groups (COGs) of proteins using blastp against the NCBI COG 2014 database [23]. Additional gene features were predicted using TMHMM Server 2.0 [24], SignalP Server (version 4.1) [25], CRISPRfinder (last update 2016–09-01) [26] and ISsaga (version 2.0) [27]. PHASTER [28] was then used to identify prophage regions in the BG1 genome. A prophage region was considered to be intact if the associated completeness score was above 90, questionable if the score was between 70 and 90 and incomplete if the score was less than 70 [28].

## Genome properties

The genome of 10.1601/nm.3093 BG1 consists of 4,782,107 bp with no discernible plasmid (no match retrieved with PlasmidFinder version 1.3 [29]), and a G + C content of 50.7%. The genome has been assembled into 84 contigs. Of the 4562 predicted genes, 4465 coded for protein and 97 were RNA-related (including eight 5S rRNA genes, suggesting the presence of 8 rRNA operons, and 86 tRNA genes). In addition, 22 pseudo genes were identified. Among the 4465 protein coding genes, 3831 (85.8%) had an assigned function while the 634 remaining genes (14.2%) encoded proteins annotated as hypothetical or unknown. In addition, the BG1 genome contained 38 predicted insertion sequences (ISs), 4 intact and 1 questionable prophage regions, and 2 CRISPR elements suggesting possible genetic crosstalk, such as horizontal gene transfer among the 10.1601/nm.3093 population. The genome properties are presented in Table 3. The distribution of genes into COGs functional categories is summarized in Table 4.

**Table 3:** Genome statistics

Attribute	Value	% of Total^a^
Genome size (bp)	4,782,107	100.00
DNA coding (bp)	4,218,785	88.22
DNA G + C (bp)	2,424,397	50.70
DNA scaffolds	84	
Total genes	4562	100.00
Protein coding genes	4465	97.88
RNA genes	97	2.13
Pseudo genes	22	0.48
Genes in internal clusters	1171	25.67
Genes with function prediction	3831	83.98
Genes assigned to COGs	3814	83.60
Genes with Pfam domains	275	6.03
Genes with signal peptides	174	3.81
Genes with transmembrane helices	1080	23.67
CRISPR repeats	2	

^a^The total is based on either the size of the genome in base pairs or the total number of proteins coding genes in the annotated genome

All the information has been obtained from Prokka annotation

**Table 4:** Number of genes associated with general COGs functional categories

Code	Value	% age^a^	Description
J	250	6.55	Translation, ribosomal structure and biogenesis
A	2	0.05	RNA processing and modification
K	293	7.68	Transcription
L	154	4.04	Replication, recombination and repair
B	0	0.00	Chromatin structure and dynamics
D	41	1.07	Cell cycle control, cell division, chromosome partitioning
V	93	2.44	Defense mechanisms
T	176	4.61	Signal transduction mechanisms
M	271	7.11	Cell wall/membrane/envelope biogenesis
N	156	4.09	Cell motility
U	60	1.57	Intracellular trafficking, secretion, and vesicular transport
O	153	4.01	Post-translational modifications, protein turnover, chaperones
C	282	7.39	Energy production and conversion
G	381	9.99	Carbohydrate transport and metabolism
E	335	8.78	Amino acid transport and metabolism
F	101	2.65	Nucleotide transport and metabolism
H	169	4.43	Coenzyme transport and metabolism
I	119	3.12	Lipid transport and metabolism
P	190	4.98	Inorganic ion transport and metabolism
Q	53	1.39	Secondary metabolites biosynthesis, transport and catabolism
R	211	5.53	General function prediction only
S	238	6.24	Function unknown
–	750	7.43	Not in COGs

^a^The total is based on the total number of proteins coding genes in the annotated genome

### Extended insights

#### Genome repertoire comparison

It is admitted that bacterial genome sequences show significant diversity due to horizontal gene transfers, gene loss and other genomic rearrangements [1]. In this report, characteristics of whole genome datasets of a selection of 10.1601/nm.3093 strains were compared with those of 10.1601/nm.3093 BG1 (Table 5). Our main objective was to compare the genome of BG1 with that of bovine (K71 and W26) and human (SE15 and Nissle) commensal 10.1601/nm.3093 strains, but we also included a bovine pathogenic strain (VL2732) and a human EHEC pathogen (Sakai), as the bovine intestine is the main reservoir of EHEC [10]. A human uropathogenic strain (10.1601/strainfinder?urlappend=%3Fid%3DNCTC+9001 ^T^), which is also the 10.1601/nm.3093 type strain, was also included as reference. These strains were assigned to different phylogroups (Additional file 1: Table S1; Fig. 2). As expected, the greatest difference in genome size was observed between BG1 and the EHEC strain Sakai (the genome size of BG1 is 812,370 bp smaller than the Sakai genome [17.0% of the BG1 genome]). This difference could be explained by the number of mobile genetic elements: the Sakai genome contains 18 prophage regions (at most 5 in the BG1 genome) and 80 insertion sequences (38 in the BG1 genome) [30]. About half of the Sakai-specific sequences are of bacteriophage origin and carry the genes involved in EHEC pathogenesis (bloody diarrhea, hemolytic uremic syndrome) [30]. More surprisingly, the chromosome length of the commensal 10.1601/nm.3093 Nissle 1917 is 659,093 bp larger than the BG1 genome (13.8% of the BG1 genome). 10.1601/nm.3093 Nissle 1917 is a human commensal strain known to be a successful colonizer of the human gut and used as a probiotic for the treatment of various intestinal disorders [31]. It is well documented that the Nissle genome carries at least three genomic islands (GEIs) inserted at different tRNA sites (*serX*, *argW* and *pheV*) probably acquired by horizontal gene transfer [32, 33]. These GEIs contained genes encoding proteins considered as fitness factors (microcins, iron uptake systems, proteases …) contributing to survival of 10.1601/nm.3093 Nissle and successful colonization of the human body [32, 33]. These GEIs were found in non-pathogenic 10.1601/nm.3093 strains but were also frequently distributed among ExPEC strains [32]. Sequence comparison showed that the genes carried by Nissle 1917 GEIs (*mch, mcm, iro, iuc, sat, iha, ybt*) are absent in the BG1 genome, suggesting the absence of these GEIs in BG1.

**Table 5:** Characteristics of whole genome datasets of different 10.1601/nm.3093 strains

Strain name	Phylo group	Origin^a^	Plasmid(s)	Genome size (bp) [chromosome + plasmid(s)]	G + C ratio (%)	CDS (nb)	Protein coding regions (nb)	rRNA operons (nb)^b^	tRNA genes (nb)
BG1	B1	Bc	0	4,782,107	50.7	4562	4465	8	86
K71	B1	Bc	0	5,115,070	50.7	5178	4872	4	65
W26	B1	Bc	0	5,118,532	50.6	4925	4852	4	66
Nissle 1917	B2	Hc	0	5,441,200	50.6	5417	4970	10	121
SE15	B2	Hc	1	4,839,683 [4,717,338 + 122,345]	50.7	4763	4572	7	85
NCTC86	A	Hc	0	5,111,920	50.6	5243	4934	7	87
VL2732	A	Bp	0	4,664,032	50.6	4615	4363	4	71
Sakai	E	Hp	2	5,594,477 [5,498,450 + 92,721 + 3306]	50.5	5447	5324	7	103
NCTC9001^T^	B2	Hp	0	5,038,133	50.6	5154	4859	6	62

^a^B: bovine; H: human; c: commensal; p: pathogen

^b^Minimal number of rRNA operons based on Prokka (BG1) or Genbank (K71, W26, VL2732, 10.1601/strainfinder?urlappend=%3Fid%3DNCTC+86, 10.1601/strainfinder?urlappend=%3Fid%3DNCTC+9001 ^T^) annotation or on *rrn*DB (version 5.1) information [69]

In accordance with the differences in genome size, the highest number of tRNA genes, described as common sites for integration of foreign DNA elements (bacteriophages, genomic islands), were detected in the genome of 10.1601/nm.3093 strains Nissle and Sakai (121 and 103 tRNA genes, respectively while only 86 were identified in the BG1 draft genome (Table 5). The genome of the remaining strains carried 62 (in the type strain 10.1601/strainfinder?urlappend=%3Fid%3DNCTC+9001 ^T^) to 85 tRNA-encoding genes (Table 5). These numbers may be slightly different depending on the annotation pipeline used for the draft genome sequences.

#### Virulence factors

The genes encoding virulence factors in the 10.1601/nm.3093 BG1 genome were analyzed using blastn against the Virulence Factors Database genomic dataset [34]. A total of 164 genes encoding virulence factors were identified in BG1 (Additional file 2: Table S2), while 181 and 202 genes encoding virulence factors were found in the reference strains 10.1601/strainfinder?urlappend=%3Fid%3DNCTC+86 and 10.1601/strainfinder?urlappend=%3Fid%3DNCTC+9001 ^T^, respectively. In-depth analysis of the BG1 genome showed that most of these genes are involved in bacterial adherence to the host epithelium, iron acquisition systems (siderophores) and flagella synthesis. As expected, genes coding for toxins produced by pathogenic 10.1601/nm.3093 strains responsible for diarrhea or intestinal damage in mammals (Shiga-toxin, heat stable [ST] toxin**,** heat-labile [LT] toxin, heat-stable enterotoxin 1 [EAST1], cytotoxic necrotizing factor 1 [CNF1]) are absent in the BG1 genome. The 10.1601/nm.3093 BG1 genome also lacks the genes encoding α-hemolysin and enterohemolysin which are involved in the virulence of pathogenic 10.1601/nm.3093 strains.

#### Adherence systems

A total of 49 genes coded for the synthesis of organelles involved in adherence of 10.1601/nm.3093 to host intestinal epithelium (Additional file 3: Table S3). Accordingly, the transmission electron micrograph of 10.1601/nm.3093 BG1 showed numerous fimbriae surrounding the bacteria (Fig. 1). Removal of partial genes and incomplete gene clusters revealed that BG1 possessed the genetic information required to encode 12 potentially functional full adherence systems (Table 6). All these systems are known to be produced by pathogenic 10.1601/nm.3093 and to adhere in vitro to different cells lines (Table 6) (for reviews see [35–37]). These adherence systems reflect the ability of commensal 10.1601/nm.3093 to colonize distinct niches during its transit through the different compartments of the bovine gastro-intestinal tract. It is also of interest to note that some of these adherence systems possess characteristics corresponding to physiological conditions encountered in the bovine gastro-intestinal tract: i) *eaeH* expression is induced at 39 °C, the internal bovine temperature, but not at 37 °C [38] ii) the pili HCP is involved in adherence of 10.1601/nm.3093 to bovine gut explants [39] and iii) the F9 fimbriae are essential for in vivo colonization of calves [40]. Furthermore, the *stg* and *F9* gene clusters are strongly associated with 10.1601/nm.3093 belonging to phylogenetic group B1 [41, 42]. To broaden these results, in silico analysis of adherence systems carried by additional 10.1601/nm.3093 strains (human and bovine commensal and pathogenic isolates) (Additional file 1: Table S1; Additional file 4: Figure S1) was also performed. A hierarchical clustering based on the presence/absence of 78 distinct adherence systems encoding genes was built using R (version 3.3.1) [43]. As shown in Additional file 4: Figure S1, bovine and human 10.1601/nm.3093 strains were not separately distributed (the closest relative strains to BG1 were the human 10.1601/nm.3093 strains S11 and IAI1 [Additional file 4: Figure S1]) suggesting that the adherence systems are associated with the adaptation of 10.1601/nm.3093 to a specific habitat (i.e. the digestive tract) rather than host specificity. As expected, the uropathogenic strain 10.1601/strainfinder?urlappend=%3Fid%3DNCTC+9001 ^T^ possesses the *pap* ACDEGHIK genes which are specific to UPEC strains [44].

**Table 6:** Adherence systems encoded by the *E. coli* BG1 genome

Adherence system	Gene or genes cluster	Pathotype^a^	in vitro cell adherence^b^	Receptor
Curli fimbriae	*csgDEFG, csgBA*	EHEC, ETEC, aEPEC, APEC	T84	Matrix, plasma proteins
EhaA autotransporter	*ehaA*	EHEC, EAEC, ETEC, AIEC, EPEC	Primary bovine epithelial cells (terminal rectum)	Unknown
EhaB autotransporter	*ehaB*	EHEC, UPEC, ETEC, EIEC, EAEC	NA^c^	Collagen I, laminin
EhaC autotransporter	*ehaC (yfaL)*	EHEC, UPEC	Unknown	Unknown
ECP (*E. coli* Common Pilus)	*ecpRABCDE*	ETEC, EHEC, NMEC, EAEC, aEPEC, septicemia	HT29, Hep-2, HeLa, HTB-4	Arabinosyl residues
ELF (*E. coli* Laminin-binding Fimbriae)	*ycbQRST*	EHEC, aEPEC	HT29, Hep-2, MDBK	Laminin
F9 Fimbriae	*z2200-z2206*	EHEC, UPEC, APEC, AIEC, EAEC, EPEC	EBL	Bovine fibronectin, Galβ1-3GlcNAc
EaeH adhesin	*eaeH*	UPEC, EHEC, ETEC, NMEC	UM-UC-3, Caco-2, CHO, HeLa, Vero	Unknown
HCP (Hemorrhagic Coli Pilus)	*hcpABC (ppdD-hofBC)*	ETEC, EHEC, aEPEC, APEC	T84, Caco-2, HeLa, Hep-2, MDBK, cow colon explants	Laminin, fibronectin
Stg fimbriae	*stgABCD*	APEC, UPEC	UM-UC-3, INT 407	Unknown
T1P (Type I pili)	*fimBE, fimAICDFGH*	UPEC, aEPEC, EAEC, APEC, STEC	HeLa, REC, colonic and ileal enterocytes	Mannose
UpaG autotransporter	*upaG*	UPEC	T24	Fibronectin, laminin

^a^See the “Abbreviations” paragraph

^b^Cell lines: T84 (human colonic adenocarcinoma), HT29 (human colorectal adenocarcinoma), Hep-2 (epithelial cells from epidermoid carcinoma of the human larynx), HeLa (human cervix epithelial carcinoma)**,** HTB-4 (human bladder transitional carcinoma), MDBK (Madin-Darby bovine kidney), EBL (embryonic bovine lung), UM-UC-3 (human bladder carcinoma), Caco-2 (human colon carcinoma), CHO (Chinese hamster ovary), Vero (kidney epithelial cells from an African green monkey), INT 407 (HeLa derivative), REC (humen B cell lymphoma), T24 (human bladder transitional carcinoma)

^c^NA: no adherence to the cells lines tested

Some of these adherence systems possess redundant properties: EhaB, ELF, HCP and UpaG are known to bind to laminin and curli, EhaA, EhaB, EhaC, ECP, F9, EaeH, HCP and UpaG are involved in biofilm formation (Table 6). This suggested an important role of both laminin binding and biofilm formation in survival and/or multiplication of commensal 10.1601/nm.3093. Laminin is an extracellular matrix commonly present in the mammalian intestine which act as an interlinking molecule in connective tissues that promote bacterial adhesion and colonization to the host tissues [45]. Moreover, commensal 10.1601/nm.3093 strains can reside in mixed biofilms in the mucus layer covering the mouse intestine [4, 46]. Because the survival of 10.1601/nm.3093 depends on anaerobes that degrade polysaccharides included in the mucus layer, it has been hypothesized that the anaerobes in the mixed biofilms provide 10.1601/nm.3093 with monosaccharide locally rather than from a mixed pool available to all species [4, 46]. Therefore, the mixed biofilm formation can results in a more efficient carbon source for commensal 10.1601/nm.3093 strains in the mammalian gut [4, 46].

As discussed above, the adhesion systems encoded by the BG1 genome were associated with 10.1601/nm.3093 strains mostly isolated from clinical cases (Table 6). However, it is important to note that the BG1 genome did not carry the genes encoding the F17, F5 and F41 fimbriae and the afimbrial adhesin CS31A mainly associated with bovine pathogenic 10.1601/nm.3093 strains involved in diarrhea [47]. For example, a recent epidemiological study showed that the F5/F41 fimbriae were prevalent among bovine diarrheagenic 10.1601/nm.3093 isolated in France [48]. The genes encoding F17, F5 and F41 are not detected in the genome of the human and bovine 10.1601/nm.3093 strains included in this study suggesting that these adherence systems are specific to bovine intestinal pathogenic 10.1601/nm.3093.

#### Flagella synthesis

A total of 47 genes encoding proteins required for flagella synthesis were present in the BG1 genome. Accordingly, the transmission electron micrograph of 10.1601/nm.3093 BG1 showed peritrichous flagella attached to the bacterial cell surface and clearly distinct from fimbriae (Fig. 1). Flagella are mainly locomotive organelles allowing bacterial movements. However, it is well documented that the flagella (also known as H-antigen) of some pathogenic 10.1601/nm.3093 mediate the adhesion to or invasion of epithelial cells (NMEC, aEPEC, ETEC, EAEC, EHEC, APEC) and contribute to biofilm formation (UPEC, ETEC) (for a review see Zhou et al. [49]). In particular, flagella of aEPEC, ETEC and EHEC strains specifically recognized a receptor located at the microvillus tips of human enterocytes [50]. Interestingly, 10.1601/nm.3093 BG1 possesses the genetic information required to encode the flagella H21, a H antigen type reported to be involved in the invasion of EHEC O113:H21 into 10.1601/strainfinder?urlappend=%3Fid%3DHCT+8 colonic epithelial cells [49]. Also, it should be noted that STEC strains with serotype O159:H21 have been isolated from bovine as well as porcine feces [51, 52].

#### Iron acquisition systems

Complete genetic information required for enterobactin synthesis (*entABCDEFS*) and ferric-enterobactin uptake (*fepABCDEFG*) was present in the genome of 10.1601/nm.3093 BG1 (Additional file 2: Table S2). Siderophores, including enterobactin, are mechanisms secreted by 10.1601/nm.3093 to scavenge iron in order to survive and multiply in hosts or external environments. Siderophores are usually described as crucial for the proliferation of pathogenic 10.1601/nm.3093 in the host and have been classified as virulence factors. However, enterobactin is frequently produced by commensal 10.1601/nm.3093 isolated from healthy mammals (human and animal isolates) [53]. *ent* and *fep* genes were also found in the genome of the reference strain 10.1601/strainfinder?urlappend=%3Fid%3DNCTC+86 (data not shown). Accordingly, Pi et al. have demonstrated that enterobactin plays a fundamental role in the colonization of healthy mouse gastro-intestinal tract by non-pathogenic 10.1601/nm.3093 [54].

#### Ethanolamine utilization

In a previous study, we demonstrated that ethanolamine present in the bovine gut is used by EHEC as a nitrogen source [11]. Furthermore, ethanolamine promotes expression of fimbrial genes and influenced EHEC adherence to epithelial cells [55]. Interestingly, 10.1601/nm.3093 BG1 is unable to degrade ethanolamine present in the bovine intestine, while the EHEC reference strain EDL933 gains a growth competitive advantage by assimilating ethanolamine in bovine intestinal content [11]. Therefore, we performed in-depth analysis of the genes involved in ethanolamine utilization in order to understand the inability of the commensal strain BG1 to use ethanolamine as a nitrogen source.

**Fig. 3:**
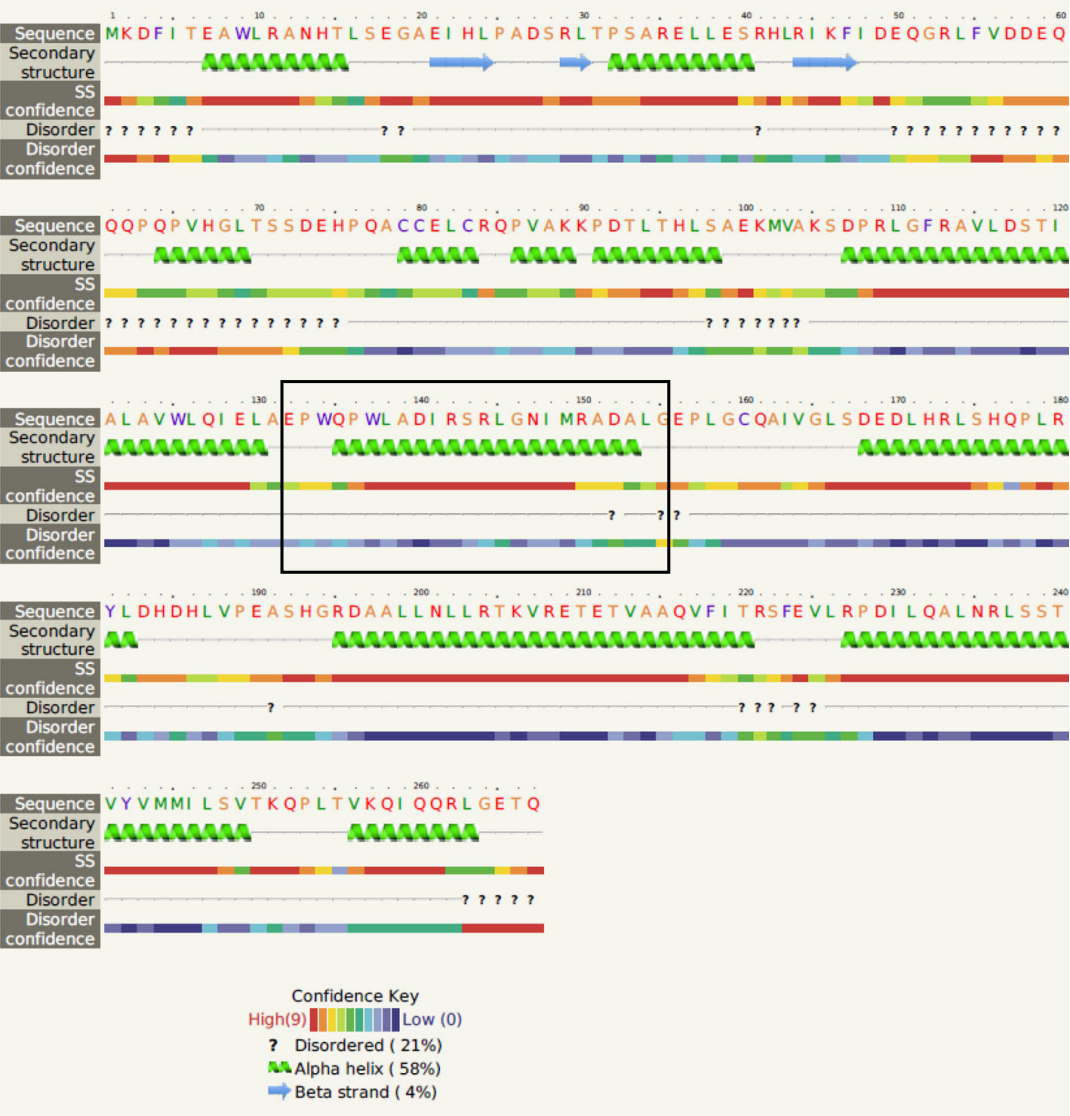
Predicted secondary structure modeling of the EutT protein of *E. coli* BG1 obtained with Phyre version 2.0 [57]

The degradation and assimilation of ethanolamine by EHEC EDL933 requires exogenous adenosylcobalamin (Ado-Cbl) and are encoded by 17 genes included in the *eut* operon [11]. In this study, we used blastn and Seaview (version 4.6.1) [56] to compare the *eut* genes of 10.1601/nm.3093 BG1 with those of EHEC EDL933. Sequence alignment showed 317 SNPs between the two *eut* operons (97.82% identity) (Additional file 5: Table S4). In addition, no premature stop codon was detected and only 34 amino acid changes due to non-synonymous SNPs were identified among the 17 predicted polypeptides encoded by the *eut* operon of BG1 (Additional file 5: Table S4). Furthermore, the presence of a 72 bp insertion was also identified in the *eutT* gene coding for cobalamin adenosyltransferase in the BG1 genome compared with the EDL933 genome (Additional file 6: Figure S2). It is important to note that ethanolamine ammonia-lyase, the key enzyme in ethanolamine degradation, required the Ado-Cbl cofactor produced by EutT to be active. The 72 bp insertion sequence at position 395 resulted in a modified translated polypeptide with 24 additional amino acids at position 132. The possible EutT conformation illustrated in Fig. 3 was predicted using Phyre (version 2.0) [57] and showed that 18 of the 24 amino acids encoded by the 72 bp sequence were predicted to form an additional alpha helix in the BG1 EutT protein.

In summary, in view of the 34 amino acid changes due to non-synonymous SNPs among the 17 predicted polypeptides encoded by the *eut* operon and the prediction of an additional alpha helix in BG1 EutT cobalamin adenosyltransferase, we suspected a reduced or abolished ethanolamine ammonialyase activity, which could explain the inability of BG1 to assimilate ethanolamine in the bovine digestive tract.

## Conclusion

The comparison of whole genomes provides information on gene content and organization, and gives an overview of how organisms are related. The draft genome sequence of 10.1601/nm.3093 BG1 isolated from the bovine intestine is now available and can provide valuable information at the genomic scale to explore the genetic and functional features adapted to the bovine gut. The genome of 10.1601/nm.3093 BG1 can be used as a reference for subsequent evolution and comparative studies (some examples of genome comparative analysis have already been described in this report).

As expected, the BG1 genome does not carry the genetic information encoding toxins responsible for intestinal damage. More surprisingly, the 10.1601/nm.3093 BG1 strain possesses the genetic information required to encode systems classified as “virulence factors” and produced by pathogenic 10.1601/nm.3093. This could suggest that genes encoding virulence factors are “in transit” from commensal species that act as genetic depositories with the ability to transmit DNA fragments to pathogenic 10.1601/nm.3093 strains. However, both pathogenic and non-pathogenic 10.1601/nm.3093 strains are able to colonize the gut and seem to use similar factors to adhere to the host epithelial cells. Therefore, it is questionable whether the ability of intestinal 10.1601/nm.3093 to colonize the host gut (resistance to the intestinal flux), excrete siderophores (iron uptake from the surrounding environment) and produce flagella (capacity to move toward nutrient-rich environments) can be considered as “virulence factors”. The terms “virulence”, “fitness” and “colonization” factors appear to overlap for 10.1601/nm.3093 species. In fact, factors contributing to 10.1601/nm.3093 survival in a given environment should be considered as fitness and adaptation factors enabling successful colonization of the host rather than strict markers of pathogenesis. In contrast, the factors responsible for disease establishment or intestinal damages during infection (e.g. aqueous or hemorrhagic diarrhea), such as toxins or the type III secretion system, appear to be true virulence factors.

## Additional files

Additional file 1: Table S1. *E. coli* strains included in this study (XLSX 13 kb)

Additional file 2: Table S2. Genes encoding virulence factors in the *E. coli* BG1 genome (XLSX 31 kb)

Additional file 3: Table S3. Genes encoding adherence systems in *E. coli* BG1 genome (XLSX 11 kb)

Additional file 4: Figure S1. Hierarchical clustering of *E. coli* strains according to adherence systems encoding genes. The dendrogram and associated heatmap are generated on the basis of gene presence/absence considering 78 genes involved in adherence, using binary distance and complete clustering method, R version 3.3.1. [43]. Blue color indicates gene presence, red gene absence. The origin of each strain is identified with B (Bovine) or H (Human). The color of the strain name corresponds to its phylogroup as in Fig. 2. (DOCX 67 kb)

Additional file 5: Table S4. Genes encoding the transport and assimilation of ethanolamine in *E. coli* BG1 genome. (XLSX 12 kb)

Additional file 6: Figure S2. Nucleotide sequence alignment of the *eutT* gene. The sequences of the *eutT* gene and the translated EutT polypeptide were aligned respectively from *E. coli* strains BG1 and EDL933 using Seaview version 4.6.1 [56]. (DOCX 15 kb)

## Abbreviations

AIEC: Adherent-invasive *E. coli*
APEC: Avian pathogenic *E. coli*
EAggEC: Enteroaggregative *E. coli*
EHEC: Enterohemorrhagic *E. coli*
EPEC: Enteropathogenic *E. coli*
ETEC: Enterotoxigenic *E. coli*
ExPEC: Extraintestinal pathogenic *E. coli*
NMEC: Neonatal meningitis *E. coli*
STEC: Shiga-producing *E. coli*
UPEC: Uropathogenic *E. coli*
