# Supplementary material for: Draft genome sequence and characterization of commensal Escherichia coli strain BG1 isolated from bovine gastro-intestinal tract
Source: Stand Genomic Sci. 2017 Oct 10;12:61. doi: 10.1186/s40793-017-0272-0 (PMC5634895; doi:10.1186/s40793-017-0272-0)
Supplement: Supplementary file 4 — Hierarchical clustering of E. coli strains according to adherence systems encoding genes. The dendrogram and associated heatmap are generated on the basis of gene presence/absence considering 78 genes involved in adherence, using binary distance and complete clustering method, R version 3.3.1. [43]. Blue color indicates gene presence, red gene absence. The origin of each strain is identified with B (Bovine) or H (Human). The color of the strain name corresponds to its phylogroup as in Fig. 2. (DOCX 67 kb) [file 40793_2017_272_MOESM4_ESM.docx]

**
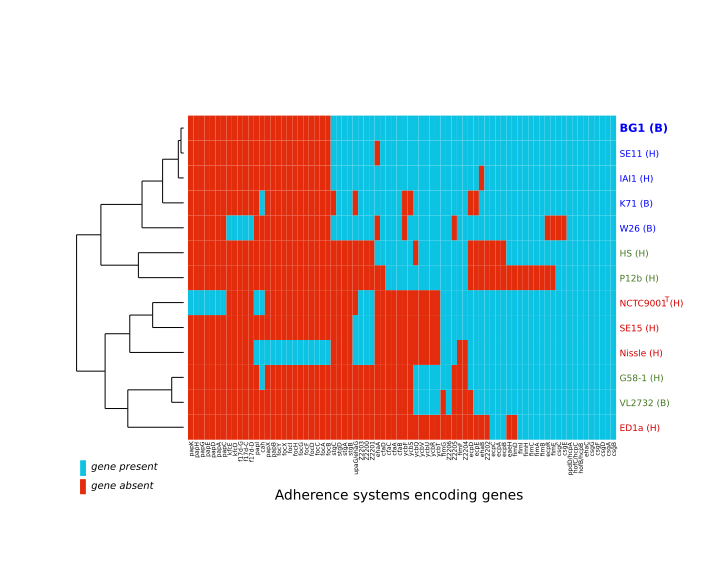
**

**Additional file 4: Fig. S1** Hierarchical clustering of *E. coli* strains according to adherence systems encoding genes.

The dendrogram and associated heatmap are generated on the basis of gene presence/absence considering 78 genes involved in adherence, using binary distance and complete clustering method, R version 3.3.1. [43]. Blue color indicates gene presence, red gene absence. The origin of each strain is identified with B (Bovine) or H (Human). The color of the strain name corresponds to its phylogroup as in Figure 2.
