# Supplementary material for: Draft genome sequence and characterization of commensal Escherichia coli strain BG1 isolated from bovine gastro-intestinal tract
Source: Stand Genomic Sci. 2017 Oct 10;12:61. doi: 10.1186/s40793-017-0272-0 (PMC5634895; doi:10.1186/s40793-017-0272-0)
Supplement: Supplementary file 6 — Nucleotide sequence alignment of the eutT gene. The sequences of the eutT gene and the translated EutT polypeptide were aligned respectively from E. coli strains BG1 and EDL933 using Seaview version 4.6.1 [56]. (DOCX 15 kb) [file 40793_2017_272_MOESM6_ESM.docx]

BG1 ATGAAAGATTTCATCACCGAAGCATGGCTAAGAGCGAACCATACGCTCAGCGAAGGCGCA 60

EDL933 ATGAAAGATTTCATCACCGAAGCATGGCTAAGAGCGAACCATACGCTCAGCGAAGGCGCA 60

BG1 M K D F I T E A W L R A N H T L S E G A 20

EDL933 M K D F I T E A W L R A N H T L S E G A 20

BG1 GAGATCCATCTCCCTGCGGACAGTCGCCTGACGCCCTCTGCCCGGGAGTTGCTGGAAAGC 120

EDL933 GAGATCCATCTCCCTGCGGACAGTCGCCTGACGCCCTCTGCCCGGGAGTTGCTGGAAAGC 120

BG1 E I H L P A D S R L T P S A R E L L E S 40

EDL933 E I H L P A D S R L T P S A R E L L E S 40

BG1 CGCCATCTGCGCATCAAGTTTATTGACGAGCAGGGCCGCCTGTTTGTTGACGATGAACAG 180

EDL933 CGCCATCTGCGCATCAAGTTTATTGACGAGCAGGGCCGCCTGTTTGTTGACGATGAACAG 180

BG1 R H L R I K F I D E Q G R L F V D D E Q 60

EDL933 R H L R I K F I D E Q G R L F V D D E Q 60

BG1 CAGCAGCCGCAGCCCGTTCATGGGCTGACCAGTAGCGATGAACATCCGCAGGCGTGCTGC 240

EDL933 CAGCAGCCGCAGCCCGTTCATGGGCTAACCAGTAGCGATGAACATCCGCAGGCGTGCTGC 240

BG1 Q Q P Q P V H G L T S S D E H P Q A C C 80

EDL933 Q Q P Q P V H G L T S S D E H P Q A C C 80

BG1 GAACTGTGTCGCCAGCCGGTGGCAAAAAAGCCGGATACGCTGACTCACCTGTCGGCGGAA 300

EDL933 GAACTGTGCCGCCAGCCGGTGGCAAAAAAGCCGGATACGCTGACTCACCTGTCGGCGGAA 300

BG1 E L C R Q P V A K K P D T L T H L S A E 100

EDL933 E L C R Q P V A K K P D T L T H L S A E 100

BG1 AAAATGGTCGCCAAAAGCGATCCGCGTCTGGGTTTTCGCGCGGTTCTCGACAGCACCATT 360

EDL933 AAAATGGTCGCCAAAAGCGATCCGCGTCTGGGTTTTCGCGCAGTTCTCGACAGCACCATT 360

BG1 K M V A K S D P R L G F R A V L D S T I 120

EDL933 K M V A K S D P R L G F R A V L D S T I 120

BG1 GCGCTGGCAGTGTGGCTGCAAATTGAACTGGCGG**AACCGTGGCAGCCGTGGCTAGCGGAT** 420

EDL933 GCGCTGGCAGTGTGGCTGCAAATTGAACTGGCGG**--------------------------** 394

BG1 A L A V W L Q I E L A **E P W Q P W L A D** 140

EDL933 A L A V W L Q I E L A **- - - - - - - - -** 131

BG1 **ATCCGTTCGCGTCTTGGCAACATTATGCGCGCCGATGCGCTGGGGG**AACCGCTGGGCTGC 480

EDL933 **----------------------------------------------**AACCGCTGGGCTGC 408

BG1  **I R S R L G N I M R A D A L G** E P L G C 160

EDL933 **- - - - - - - - - - - - - - -** E P L G C 136

BG1 CAGGCGATTGTCGGGCTTTCTGACGAAGATCTGCATCGGCTTTCTCATCAGCCGCTGCGC 540

EDL933 CAGGCGATTGTCGGGCTTTCTGACGAAGATCTGCACCGGCTTTCTCATCAGCCGTTGCGT 468

BG1 Q A I V G L S D E D L H R L S H Q P L R 180

EDL933 Q A I V G L S D E D L H R L S H Q P L R 156

BG1 TATCTCGACCACGATCATCTGGTGCCGGAAGCCAGCCACGGGCGCGATGCGGCGTTGCTC 600

EDL933 TATCTTGGTCACGATCATCTGGTGCCGGAAGCAAGCCACGGGCGCGATGCGGCGTTGCTG 528

BG1 Y L D H D H L V P E A S H G R D A A L L 200

EDL933 Y L G H D H L V P E A S H G R D A A L L 176

BG1 AATCTGCTACGTACCAAAGTGCGGGAAACCGAAACCGTAGCGGCGCAGGTGTTTATCACC 660

EDL933 AATCTGCTACGCACCAAAGTGCGGGAAGCCGAAACCGTAGCGGCGCAGGTGTTTATCACC 588

BG1 N L L R T K V R E T E T V A A Q V F I T 220

EDL933 N L L R T K V R E A E T V A A Q V F I T 196

BG1 CGCAGCTTTGAAGTATTACGCCCGGACATTTTGCAGGCGCTGAACCGCCTTTCGAGCACG 720

EDL933 CGCAGTTTTGAAGTATTACGCCCGGACATCTTGCAGGCGCTGAACCGCCTTTCCAGCACG 648

BG1 R S F E V L R P D I L Q A L N R L S S T 240

EDL933 R S F E V L R P D I L Q A L N R L S S T 216

BG1 GTCTACGTGATGATGATCCTGAGCGTAACGAAGCAGCCGCTAACGGTGAAGCAGATCCAA 780

EDL933 GTCTACGTGATGATGATCCTGAGCGTAACGAAGCAGCCGCTAACGGTGAAGCAGATCCAA 708

BG1 V Y V M M I L S V T K Q P L T V K Q I Q 260

EDL933 V Y V M M I L S V T K Q P L T V K Q I Q 236

BG1 CAACGACTGGGGGAAACGCAATGA 804

EDL933 CAACGACTGGGGGAAACGCAATGA 732

BG1 Q R L G E T Q * 267

EDL933 Q R L G E T Q * 243

**Additional file 6: Fig. S2** Nucleotide sequence alignment of the *eutT* gene.

The sequences of the *eutT* gene and the translated EutT polypeptide were aligned respectively from *E. coli* strains BG1 and EDL933 using Seaview version 4.6.1 [56].
